# ^68^Ga-NOTA PET imaging for gastric emptying assessment in mice

**DOI:** 10.1186/s12876-021-01642-7

**Published:** 2021-02-13

**Authors:** Xueyan Chen, Yu Liu, Donghui Pan, Maoyu Cao, Xinyu Wang, Lizhen Wang, Yuping Xu, Yan Wang, Junjie Yan, Juan Liu, Min Yang

**Affiliations:** 1 Department of Veterinary Medicine, Southwest University, Rongchang, Chongqing, 402460 China; 2 NHC Key Laboratory of Nuclear Medicine, Jiangsu Key Laboratory of Molecular Nuclear Medicine, Jiangsu Institute of Nuclear Medicine, Wuxi, 214063 Jiangsu China; 3 Immunology Center, Medical Research Institute of Southwest University, Rongchang, Chongqing, 402460 China

**Keywords:** Gastric emptying, Positron emission tomography, [^68^Ga]Ga-NOTA, Constipation, Traditional Chinese medicine

## Abstract

**Background:**

Positron emission tomography (PET) has the potential for visualization and quantification of gastric emptying (GE). The traditional Chinese medicine (TCM) has been recognized promising for constipation. This study aimed to establish a PET imaging method for noninvasive GE measurement and to evaluate the efficacy of a TCM on delayed GE caused by constipation using PET imaging.

**Methods:**

[^68^Ga]Ga-NOTA was synthesized as the tracer and sesame paste with different viscosity were selected as test meals. The dynamic PET scans were performed after [^68^Ga]Ga-NOTA mixed with test meals were administered to normal mice. Two methods were utilized for the quantification of PET imaging. A constipation mouse model was treated with maren chengqi decoction (MCD), and the established PET imaging scans were performed after the treatment.

**Results:**

[^68^Ga]Ga-NOTA was synthesized within 20 min, and its radiochemical purity was > 95%. PET images showed the dynamic process of GE. %ID/g, volume, and total activity correlated well with each other. Among which, the half of GE time derived from %ID/g for 4 test meals were 3.92 ± 0.87 min, 13.1 ± 1.25 min, 17.8 ± 1.31 min, and 59.7 ± 3.11 min, respectively. Constipation mice treated with MCD showed improved body weight and fecal conditions as well as ameliorated GE measured by [^68^Ga]Ga-NOTA PET.

**Conclusions:**

A PET imaging method for noninvasive GE measurement was established with stable radiotracer, high image quality, and reliable quantification methods. The efficacy of MCD on delayed GE was demonstrated using PET.

## Background

Gastrointestinal motility disorders such as gastroparesis, functional dyspepsia, and constipation, affect the life quality of a huge population worldwide and cause considerable medical expenses and economic burden [1, 2]. Gastric emptying (GE) is one of the most important parts of gastrointestinal motility. Since accelerated, normal, and delayed GE cannot be reliably differentiated based on specific symptoms, objective measurement of GE is required [3]. Besides, abnormal GE frequently occurs in certain diseases such as diabetes, critically ill, systematic sclerosis, and Parkinson’s disease, and the altered GE may be an important mechanism for some effective medications [4–10]. Therefore, it is important to visualize and quantify GE.

At present, accurate GE measurement remains challenges. The indirect methods such as ^13^C breathe test and paracetamol absorption test require breathe or blood analysis. These methods can be disrupted by hyperglycemia and impaired liver or kidney functions [3, 11, 12]. Conventional imaging modalities such as ultrasonography, bioluminescence, and magnetic resonance imaging (MRI) are limited by temporal or spatial resolution, quantitative capability, or low availability [13–15]. Some alternative methods were put forward in recent years and showed potential for the effective visualization and localization of solid dosage forms in the intestinal tract of rats [16]. Scintigraphy, a functional imaging modality combined with radiotracers (e.g. [^99m^Tc]Tc-phytate, [^99m^Tc]Tc-DTPA, and [^99m^Tc]Tc-sulphur colloid), is currently the reference standard for noninvasive GE evaluation which can provide direct and comprehensive visualization for GE [17].

Although scintigraphy has been widely recognized and standardized [18], few studies explored the value of positron emission tomography (PET), another functional imaging modality using radiotracers, for GE measurement. The imaging manners of PET also makes it have the advantages of scintigraphy. They are both direct measures for functional information. Both of them can visualize and determine the intragastric distribution. Besides, PET has better quantification capability and image quality and is considered to be one of the most advanced modalities for medical imaging [19–21]. Previous studies have demonstrated the potential of PET for investigating gastrointestinal absorption and pharmacokinetic profiles of drugs [22–24]. However, no PET tracer has been tested for the direct GE measurement. Perhaps the primary concern for PET is the low cost-effectiveness, but this concern can be mitigated by using more cost-effective PET radionuclides. At present, PET usually uses ^18^F as the radionuclide which is produced by the cyclotron. Just like ^99m^Tc, ^68^Ga is produced by the generator, which has the advantages of simple operation, low economic and site burdens, and no need for additional radiation shielding and high-skilled operators for the cyclotron [25]. Therefore, ^68^Ga has been currently one of the most popular PET radionuclides in both basic research and clinical applications.

Constipation has negative impacts on life quality and is a considerable burden on health-care resources. The median prevalence of constipation is > 16% worldwide. Besides, constipation is usually associated with other gastrointestinal disorders and increased prevalence of psychological distress [26, 27]. In recent years, research on the treatment of constipation using traditional Chinese medicine (TCM) has increased significantly and shown promising results [28, 29]. Maren Chengqi decoction (MCD) is a modified Chinese herbal formula based on Chengqi decoction. Chengqi decoction is a well-known TCM that showed efficacy for the treatment of gastrointestinal disorders in Chinese research [30–33]. Maren (seeds of *Cannabis sativa* L.) has the effects of laxative and is usually used for the treatment of blood deficiency and constipation in TCM [34–36]. However, it remains unclear whether these TCMs can ameliorate GE in constipation and other gastrointestinal disorders. In this study, the primary aim was to establish an available PET imaging method for accurate GE measurement using radionuclide ^68^Ga. Then, we would like to evaluate the improvement of MCD on delayed GE caused by constipation using this established PET imaging method.

## Methods

### Animals and generals

ICR mice used in this study (8–10 weeks age, half male and half female) were purchased from Changzhou Cavens Animal Co., Ltd. and housed in the Laboratory Animal Center of Jiangsu Institute of Nuclear Medicine under a temperature-controlled (23 °C ± 2 °C) and humidity-controlled (~ 50%) condition. Mice were free to access standard autoclaved rodent food [37] and water and were maintained in a 12-h light/dark cycle. All animal experiments were approved by the Laboratory Animal Management and Ethics Committee of Jiangsu Institute of Nuclear Medicine (Ethical Committee Number: JSINM-2018-043). After the experiments, mice were euthanized by cervical dislocation. All reagents, unless otherwise stated, were purchased from commercial suppliers and used without further purification. ^68^Ga^3+^ for the radiolabeling was obtained from ^68^Ge/^68^Ga generators (ITG, Germany). [^99m^Tc]Tc-DTPA which had satisfied the clinical requirement was generously provided by the Department of Nuclear Medicine, Jiangyuan Hospital. All experiments were performed blindly among researcher.

### Preparation of [^68^Ga]Ga-NOTA

The fresh ^68^Ga^3+^ was obtained in the form of [^68^Ga]GaCl_3_ which was eluted from ^68^Ge/^68^Ga generators with 4 mL 0.05 M HCl. Among them, the 1.5 mL fraction with the highest radioactivity (~ 370 MBq) was used for further radiolabeling. Then, 94 μL 1 M sodium acetate buffer was added into [^68^Ga]GaCl_3_ solution, followed by 50 μg maleimide-NOTA (CheMatech, France), and the mixture was heated at 97 °C for 10 min. The product was captured on a C18 column (Agilent, USA) using 10 mL deionized water and then eluted by 0.3 mL 10 mM HCl-containing ethanol. The radiochemical purity and in vitro stability in simulated gastric fluid [38] of the final product were measured by a radio-HPLC (Waters, USA) based on the protocol of previous studies [39].

### Preparation of test meals

We prepared 4 types of test meals to determine the feasibility of [^68^Ga]Ga-NOTA PET for GE evaluation. These test meals could represent liquid food (G1: 2 mL saline), semi-fluid food (G2: 0.5 g black sesame paste with 2 mL saline), fluid food (G3: 1 g black sesame paste with 2 mL saline), and semi-solid food (G4: 1.5 g black sesame paste with 2 mL saline), respectively. The edible black sesame paste (Nanfang, China) we used in this study was in line with Chinese dietary habits and made up of rice, black sesame (≥ 21.5%), sesame, maltodextrin, xylitol, walnuts, and peanuts. Each 40 g of them contained 696 kJ energy, 3.6 g protein, 5.2 g fat, 26 g carbohydrates, 1.8 g sugar, 40 mg sodium, 60 mg calcium, and 0.6 mg iron. The viscosity of these test meals was measured by a Rheometer (HAAKE, Germany). Finally, [^68^Ga]Ga-NOTA or [^99m^Tc]Tc-DTPA was added into these test meals before PET or scintigraphy scans.

### PET scans

All PET scans were performed on an Inveon microPET scanner (Siemens Medical Solutions, Germany). Mice were fasted for at least 12 h before scans. Since previous studies demonstrated that anesthesia might have non-negligible effects on the gastrointestinal motility of mice, the mice under conscious state were immobilized in a small-animal retainer on the scanning bed during scans [23]. Meanwhile, a dedicated observer always paid attention to the conditions of the mice, and the scans would be terminated immediately if the mice showed instability (rapid/weak breathing, limb convulsions, etc.). The four test meals (0.3 mL) were mixed with 2.59 MBq [^68^Ga]Ga-NOTA and intragastrically administered to the mice (n = 4/group) randomly at a constant rate within 10 s. The dynamic image acquisitions were continued from the beginning of the administration to 60 min after administration. The dynamic data of each scan was sorted into 53 frames (5 s × 6, 2 s × 30, 30 s × 3, 90 s × 8, 300 s × 3, 600 s × 3). Images were reconstructed using 3D-ordered subset expectation maximization.

### Image data analysis

We utilized two methods for the quantification of PET images. For the first method, regions of interest (ROIs) for the entire stomach were manually drawn on the averaged dynamic PET image of each mouse using ASIPro version 6.8. Then these ROIs were used to extract time-activity curves (TAC) of all dynamic images of each mouse and the radioactivity in ROIs was represented by image-derived percentage injected dose per gram (%ID/g). The half of GE time (T_1/2_) was defined as the reduction in intragastric radioactivity to half of the highest radioactivity on the TAC at this time point. The GE rate at any time point (GERt) was calculated as $${\text{GERt}} = \left( {1 - {\raise0.7ex\hbox{${\% {\text{ID/gt}}}$} \!\mathord{\left/ {\vphantom {{\% {\text{ID/gt}}} {\% {\text{ID/g}}(\max )}}}\right.\kern-\nulldelimiterspace} \!\lower0.7ex\hbox{${\% {\text{ID/g}}(\max )}$}}} \right)*100\%$$ and %ID/g(t) represented the radioactivity at specific time points while %ID/g(max) represented the highest radioactivity on the TAC. For the second method, reconstructed images were processed by PMOD software version 3.8 to accurately measure the volume of gastric content and calculate the total radioactivity. The 3D volumes of interest (VOIs) in stomach of each mouse was automatically extracted using PBAS module, then the average volume and PET unit values could be obtained. The total activity could be calculated as total activity = average PET unit value * conversion factor * 0.037 * volume. Then T_1/2_ and GER were calculated based on total activity and volume, respectively. Finally, we would evaluate the linear correlations between the quantified results obtained from PMOD and ASIPro.

### CT scans

For better visualization and anatomical matching of PET images, we performed micro-computed X-ray tomography (microCT) scans for these mice. Mice were anesthetized with 1.5–2.5% isoflurane/oxygen mixture before scanning and the anesthesia was maintained during the scans. The mice were lying prone on the scanning bed and the posture remained the same as that of the PET scans. All scans were performed at a tube voltage of 40 kV and a current of 200 μA. CT scans were performed from the tip of the noses to distal femurs in order to capture the entire mice in the optimal field of view. We manually compared the acquired CT images and selected one frame that best fitted the position and posture of PET scans. Finally, we manually overlaid the PET images onto the selected CT image.

### Biodistribution

The mice were sacrificed at 1 h after intragastric administration of [^68^Ga]Ga-NOTA mixed with 4 test meals (n = 4/group). The administered dose of radiotracer was the same in different groups. The samples of major tissues and organs including blood, heart, liver, spleen, lung, kidney, stomach, duodenum, colon, bladder, pancreas, muscle, and bone were dissected and weighed. The radioactivity of each sample was measured by a γ-counter and the quantification of these radioactive uptakes was expressed in %ID/g.

### Scintigraphy scans

We performed scintigraphy scans for the mice on a clinical SPECT scanner (Philips, Netherlands) in Jiangyuan Hospital. In brief, the fasted mice were immobilized on the scanning bed under conscious state. The prepared liquid test meals (0.3 mL) were mixed with 3.7 MBq [^99m^Tc]Tc-DTPA and intragastrically administered to the mice (n = 5) at a constant rate within 10 s, and then 30-min dynamic image acquisitions were conducted immediately with images acquired 1 frame/min. While the mice were intragastrically administered, we placed a cylindrical bottle with 0.3 mL liquid test meals mixed with 3.7 MBq [^99m^Tc]Tc-DTPA on the upper right side of the mice as a reference. So that we could have intuitive comparisons between the radioactivity in the stomach and the standard. ROIs were drawn manually and the total radioactive counts in these ROIs were calculated.

### Mouse model of constipation

Fifty mice were randomly selected for the induction of constipation model, and another ten mice were selected as the healthy control group. According to the TCM methods and previous studies [40, 41], the constipation model was induced by a kind of Chinese herbal decoction combined with loperamide hydrochloride (Janssen, China) as well as a moderate restriction of water. Briefly, the volatile oil of dried ginger, cinnamon, evodia rutaecarpa, and pepper were extracted. Meanwhile, the aconite was boiled with water for 30 min, then the residue of the extracted volatile oil was added and boiled for another 30 min. The product was filtered and the filtered liquid was repeatedly boiled and filtered twice for 30 min each time. The final filtrate was concentrated to ~ 1 g/mL using a rotary evaporator (IKA, Germany) and then mixed with the extracted volatile oil which was dissolved in Tween-80 (1:2). The dose for constipation induction was 2 mL/100 g body weight (i.e., 2 g herbal decoction and 0.2 mg loperamide hydrochloride) once daily for 10 days. The control group was administered with 0.9% saline at the same dose once daily for 10 days. The body weight, fecal quantity, and fecal moisture of mice were measured every day. Fecal granules of mice within 12 h after gavage were taken out and counted. Then, the feces were weighed before (wet weight) and after (dry weight) drying using an oven. The fecal moisture (%) = (wet weight − dry weight) * 100%/wet weight.

### Treatment protocol of MCD

The preparation of MCD was based on the record of TCM [31, 32]. In brief, mirabilite (a mineral containing Na_2_SO_4_·10H_2_O), rhubarb (*Rheum palmatum* L.), maren, zhishi (incompletely mature fruit of *Citrus aurantium* L.), and magnolia officinalis, were prepared according to the ratio of 4:2:2:1:1. Firstly, magnolia officinalis, zhishi, and maren (crushed) were mixed and boiled with water for 60 min and then filtered. The rhubarb was added to the filtration and boiled for another 30 min, and the mirabilite was finally added. The products were separately concentrated to 1.6 g/mL (high dose), 0.8 g/mL (middle dose), and 0.4 g/mL (low dose) using the rotary evaporator. The constipation mice that were successfully modeled were randomly divided into 5 groups immediately (n = 10/group), and 3 groups of them were given high dose, middle dose, and low dose of MCD, respectively. Another 2 groups were given 4% magnesium sulfate (positive control group) and 0.9% saline (model group), respectively. The 10 mice of healthy control were given 0.9% saline (control group) during the treatment period, too. The dose for all 6 groups was 1 mL/100 g body weight once daily for 5 days. The body weight, fecal quantity, and fecal moisture of mice were measured every day. [^68^Ga]Ga-NOTA PET dynamic scans using liquid test meals were performed after the treatment was finished immediately.

### Statistical analysis

The data were presented as mean ± SD. Statistical analysis was performed using Stata/SE 12.0. The differences between groups were analyzed by student t-test and one-way ANOVA with Bonferroni post-hoc test. Pearson’s correlation coefficient was used to evaluate the association between radioactivity calculated by ASIPro and PMOD. Results were considered statistically significant as *p* < 0.05.

## Results

### Radiochemistry and in vitro stability

**Fig. 1:**
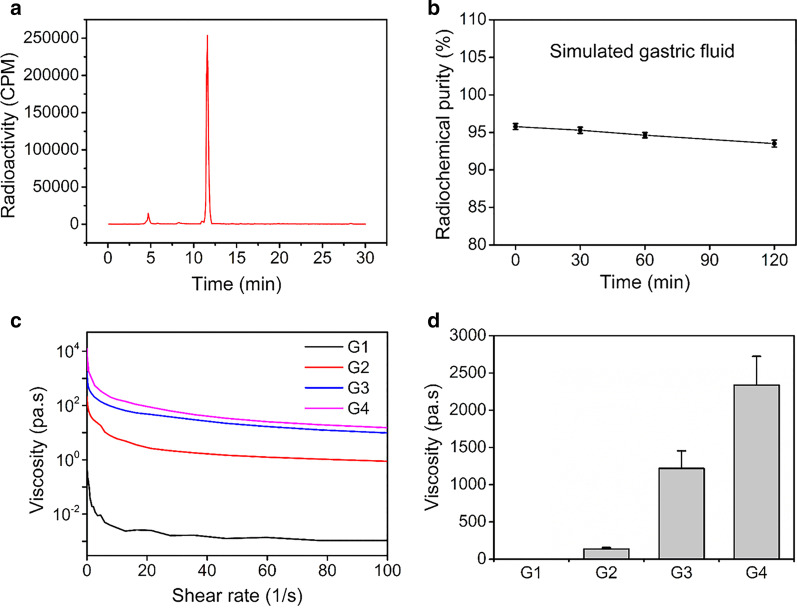
Characteristics of [^68^Ga]Ga-NOTA and test meals. **a** Radio-HPLC results of [^68^Ga]Ga-NOTA. **b** In vitro stability of [^68^Ga]Ga-NOTA in simulated gastric fluid. **c** Changes in the viscosity of 4 test meals. **d** The viscosity of test meals when shear rate near infinitesimal

The synthesis of [^68^Ga]Ga-NOTA was completed within 20 min with a radiochemical purity > 95% (Fig. 1a). The retention times of ^68^Ga^3+^ and [^68^Ga]Ga-NOTA in HPLC were approximately 4.7 and 11.6 min, respectively. After incubated in simulated gastric fluid for 2 h, the radiochemical purity of [^68^Ga]Ga-NOTA was still higher than 90% (Fig. 1b).

### Test meals

The viscosity of 4 test meals decreased with increasing shear rates (Fig. 1c). At room temperature, when the shear rate was about 0.091 1/s (near infinitesimal), the viscosity of each test meal were 0.26 ± 0.05 Pa.s (G1), 0.137 × 10^3^ ± 0.020 × 10^3^ Pa.s (G2), 1.22 × 10^3^ ± 0.238 × 10^3^ Pa.s (G3), and 2.34 × 10^3^ ± 0.387 × 10^3^ Pa.s (G4), respectively (Fig. 1d).

### PET images and quantification

**Fig. 2:**
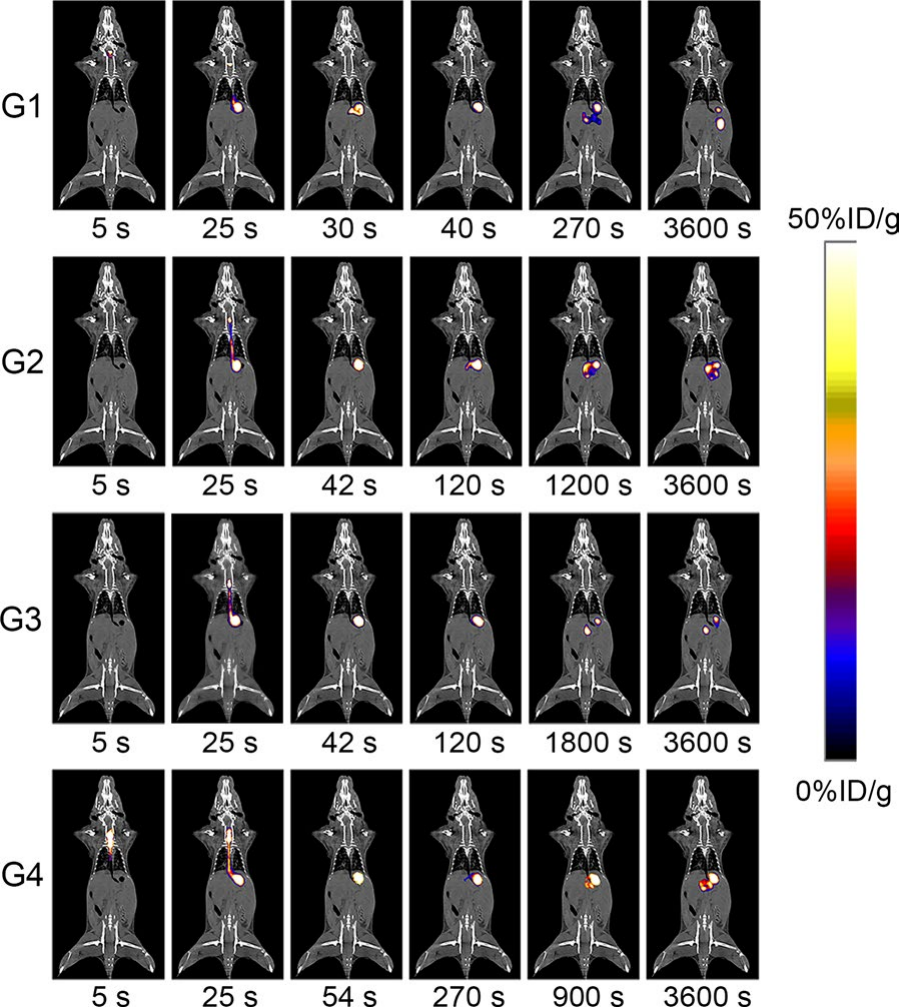
Representative PET images of 4 groups of mice at different time points after [^68^Ga]Ga-NOTA administration

The dynamic accumulation and emptying process of 4 test meals in the stomach was presented on the PET images (Fig. 2). Although the 4 groups of mice reached the maximum radioactivity in the stomach at similar time points by gavage, the PET images showed that the time points at which different contents were discharged from the stomach were significantly different. At 30 s after administration, the duodenum in the G1 group showed radioactive uptake, while the radioactivity in the G2 and G3 groups appeared to be confined to the stomach at 42 s and the G4 group still could not see the test meal discharged from the stomach at 270 s. At 1 h after administration, the radioactivity with test meal in the stomach of the G1 group was almost completely emptied, however, the tracer of the G4 group still had lots of accumulation in the stomach.

**Fig. 3:**
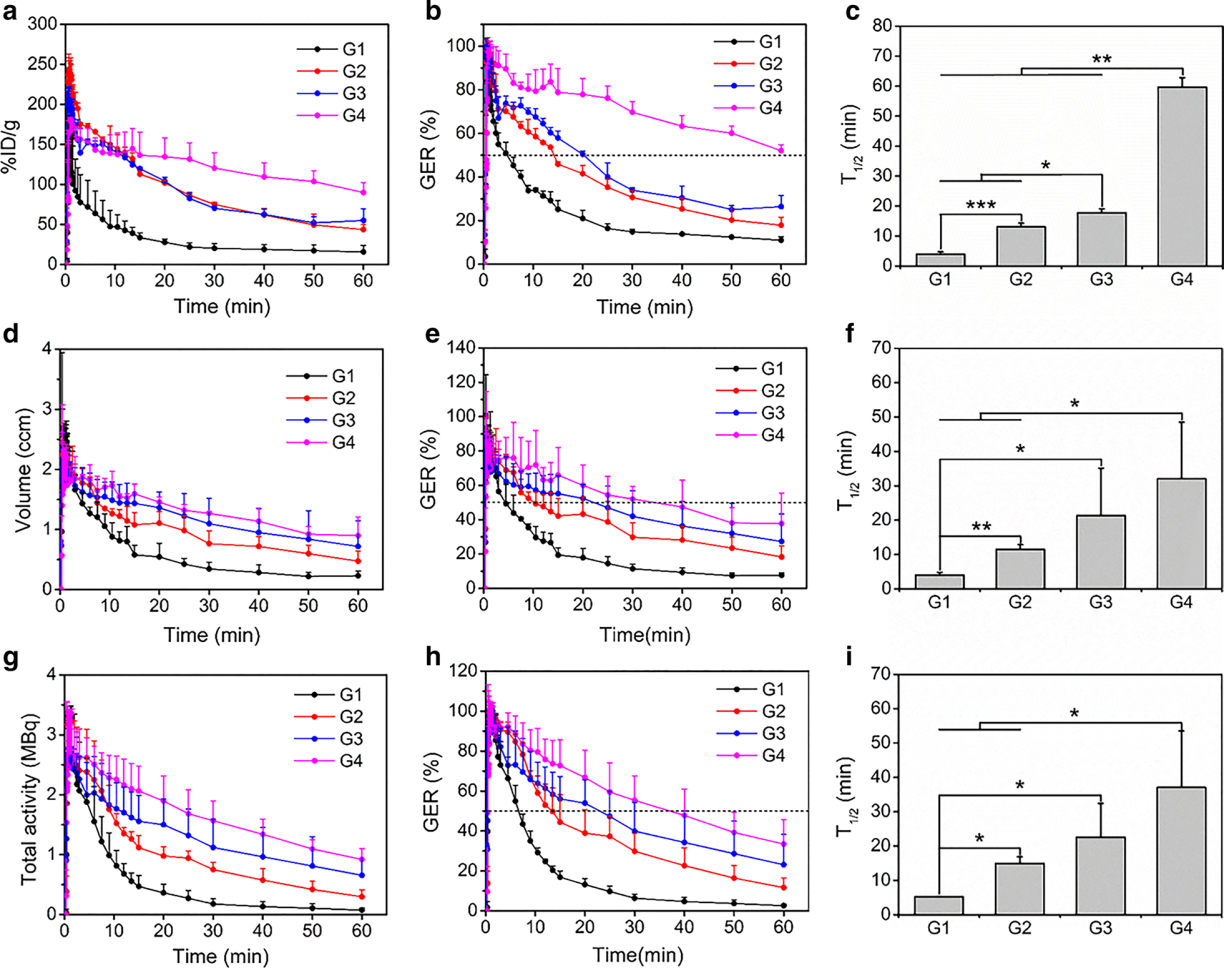
Quantification of PET images in 4 groups of mice (n = 4/group). The %ID/g (**a**), GER (**b**), and T_1/2_ (**c**) calculated by manually delineated ROIs. The volume (**d**) and total activity (**g**) and their derived GER (**e**, **h**) and T_1/2_ (**f**, **i**). *p < 0.05, **p < 0.01, ***p < 0.001, and *p* values in **c**, **f**, and **i** were calculate by one-way ANOVA with Bonferroni post-hoc test. *GER* gastric emptying rate

As the viscosity of the test meal increased, the radiotracer remained in the stomach longer (Fig. 3a, Additional file 1: Figure S1A), and the GER calculated based on %ID/g was lower (Fig. 3b, Additional file 1: Figure S1D). Figure 3c showed significant differences in T_1/2_ between any two test meals and the quantitative T_1/2_ were 3.92 ± 0.87 min, 13.1 ± 1.25 min, 17.8 ± 1.31 min, and 59.7 ± 3.11 min for G1, G2, G3, and G4, respectively. Furthermore, PMOD was used to calculate the dynamic changes in the volume and total radioactivity of gastric contents during PET imaging. Similarly, all test meals rapidly reached their maximum volume and radioactivity in the stomach and had almost the same maximum radioactivity (Fig. 3d, g, Additional file 1: Figure S1B, C). However, the time points at which the volume and radioactivity began to decrease in the stomach and their downward trends within 1 h were different among 4 groups. It was shown that liquid meal (G1) had the fastest emptying process of content volume and total activity while the downward trends were closer to the linear form with the increase of the viscosity of test meals. The changes in GER calculated by volume and radioactivity within 1 h were the same as their corresponding indexes. Besides, the T_1/2_ based on volume and total activity both showed significant differences among these test meals (Fig. 3e, f, h, i, Additional file 1: Figure S1E, F).

**Fig. 4:**
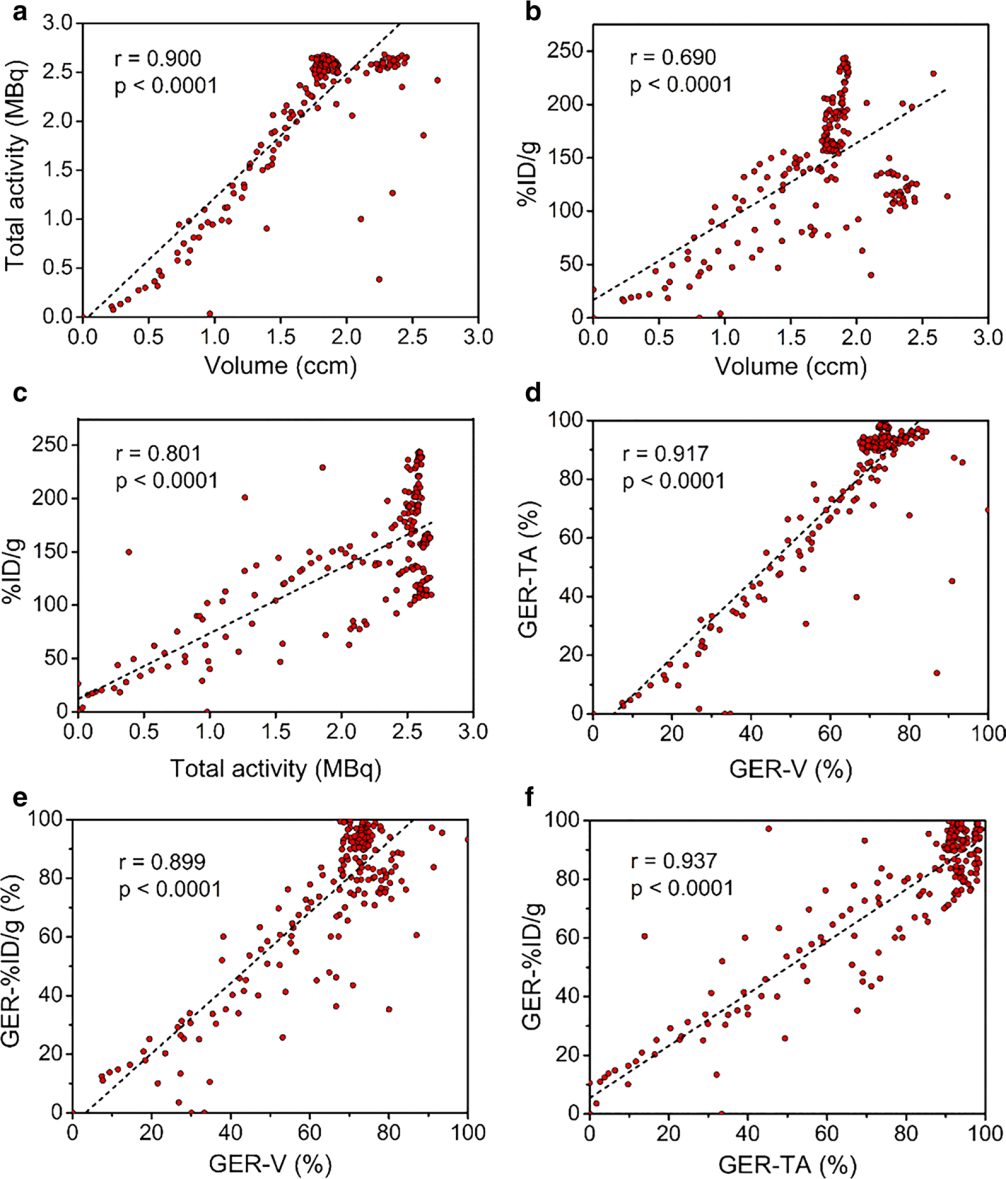
Correlations among %ID/g, total radioactivity, volume, and their derived GER. Correlations in **a**–**f** were analyzed using Pearson’s correlation coefficient. *GER* gastric emptying rate, *TA* total radioactivity, *V* volume

Finally, we analyzed the correlations among %ID/g, volume, and total activity (Fig. 4). Whether it was the dynamic changes of the 3 parameters (Fig. 4a–c) or the GERs calculated by them (Fig. 4d–f), it all showed high correlations between them (*p* < 0.0001), indicating that the %ID/g calculated using the simpler manually delineated ROIs could substitute the volume or total activity calculated by PMOD to reflect the GE process. Besides, the excellent correlation between volume and total activity demonstrated the uniform distribution of the radiotracer in the test meals.

### Biodistribution

At 1 h after administration, the radioactivity mainly accumulated in the stomach and duodenum (Table 1). With the increase of the viscosity of test meals, there was significantly higher radioactivity in the stomach and duodenum (*p* < 0.05), indicating the slower GE process in the group with higher viscosity of test meals. Besides, the radioactivity in the blood of all mice was extremely low, demonstrating the promising in vivo stability of [^68^Ga]Ga-NOTA in the gastric environment.

**Table 1:** Biodistribution of [^68^Ga]Ga-NOTA in the mice at 60 min after administration (n = 4/group)

	G1 (%ID/g)	G2 (%ID/g)	G3 (%ID/g)	G4 (%ID/g)
Blood	0.23 ± 0.06	0.22 ± 0.11	0.31 ± 0.04	0.29 ± 0.10
Heart	0.31 ± 0.20	0.35 ± 0.14	0.57 ± 0.34	0.50 ± 0.44
Liver	0.26 ± 0.10	0.19 ± 0.08	0.27 ± 0.13	0.26 ± 0.14
Spleen	0.32 ± 0.19	0.63 ± 0.35	0.62 ± 0.10	0.73 ± 0.62
Lung	0.26 ± 0.10	0.41 ± 0.40	0.35 ± 0.08	0.81 ± 0.55
Kidney	0.32 ± 0.14	0.25 ± 0.13	0.43 ± 0.21	0.67 ± 0.32
Stomach*	11.4 ± 2.20	34.6 ± 19.4	42.3 ± 16.0	85.2 ± 33.3
Duodenum	3.04 ± 2.58	6.24 ± 5.89	11.4 ± 5.20	13.5 ± 5.83
Colon	0.23 ± 0.11	1.21 ± 1.57	1.39 ± 1.41	0.52 ± 0.24
Bladder	1.26 ± 0.91	1.34 ± 0.72	1.94 ± 0.59	2.07 ± 0.76
Pancreas	0.25 ± 0.13	0.29 ± 0.08	0.35 ± 0.17	0.31 ± 0.14
Muscle	0.70 ± 0.79	0.50 ± 0.67	0.73 ± 0.44	0.59 ± 0.40
Bone	0.59 ± 0.34	0.63 ± 0.37	0.68 ± 0.39	0.58 ± 0.36

Data were represented as mean ± SD

^*^p < 0.05 among the 4 groups calculated by one-way ANOVA

### Scintigraphy scans

After intragastric administration of [^99m^Tc]Tc-DTPA mixed with liquid test meal, the total radioactive counts in the stomach acquired from scintigraphy decreased rapidly and reached equilibrium at 15 min (Additional file 2: Figure S2), and these results were similar to those shown by [^68^Ga]Ga-NOTA PET. Due to the limited image quality and quantification capability, we didn’t calculate the GER and T_1/2_ measured by scintigraphy.

### MCD ameliorated constipation symptoms

**Fig. 5:**
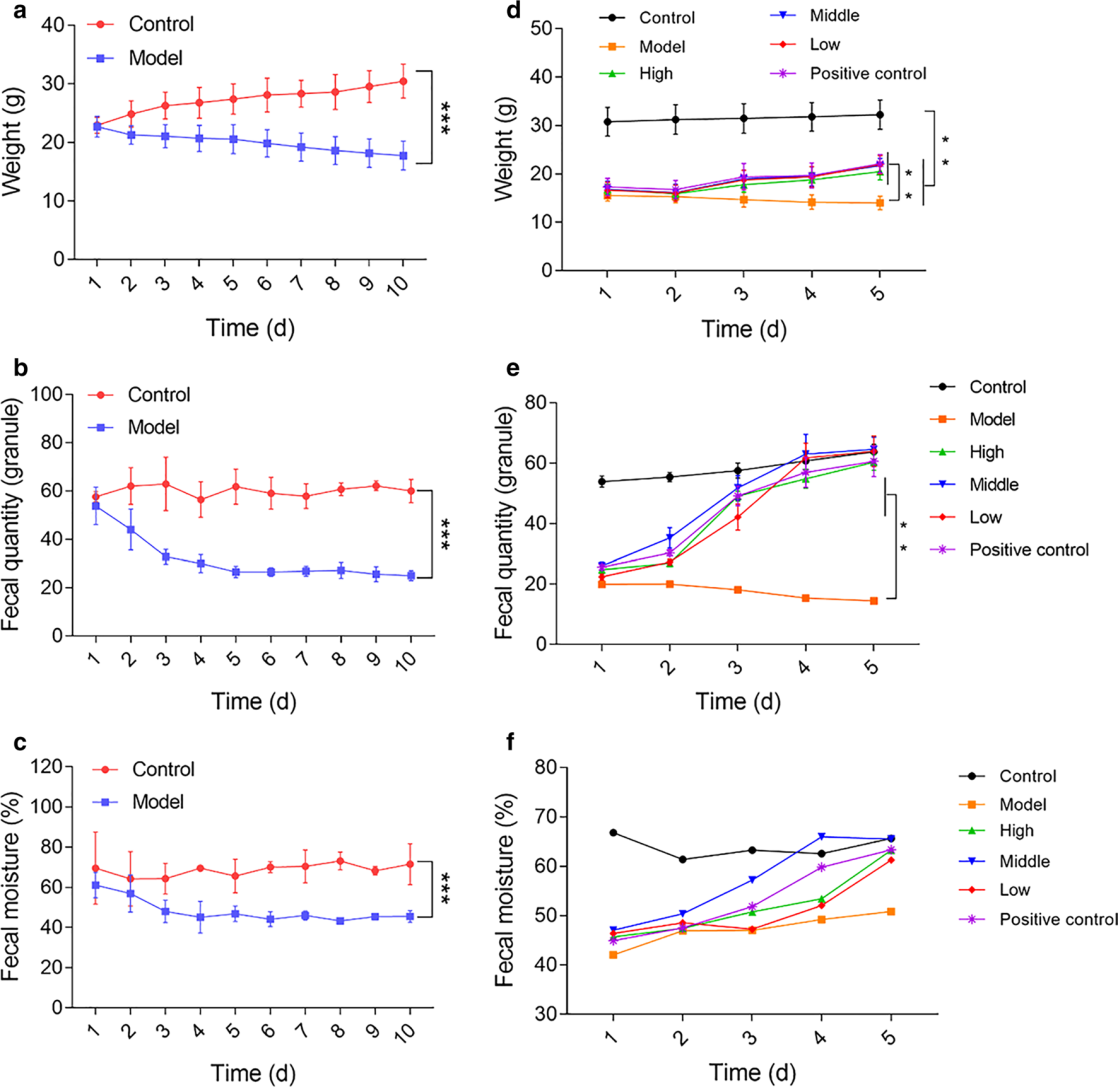
MCD ameliorated constipation symptoms. The changes in body weight (**a**), fecal quantity (**b**), and fecal moisture (**c**) of constipation model group and control group during modeling period (n = 10/group). The changes in body weight (**d**), fecal quantity (**e**), and fecal moisture (**f**) of 6 groups during MCD treatment period (n = 10/group). **p < 0.01, ***p < 0.001. Data in **b**, **c**, and **e** was from 3 individual experiments. Data in **f** was from 1 experiment. *p* values in **a**–**c** were calculated by student t-test, and *p* values in **d** and **e** were calculated by one-way ANOVA with Bonferroni post-hoc test

As shown in Fig. 5a–c and Additional file 3: Figure S3A–D, the body weight, fecal quantity, and fecal moisture of constipation mice decreased significantly during modeling period. On the contrary, the fecal quantity and moisture of control mice remained stable and their body weight increased significantly. On the day 10 of modeling, there were significant differences in body weight, fecal quantity, and fecal moisture between these two groups (*p* < 0.001). After 5 days of treatment with different doses of MCD and magnesium sulfate, the body weight, fecal quantity, and fecal moisture of constipation mice increased obviously, which was significantly different from those of untreated constipation mice (Fig. 5d–f, Additional file 3: Figure S3E–G).

### PET evaluation of MCD therapy for constipation

**Fig. 6:**
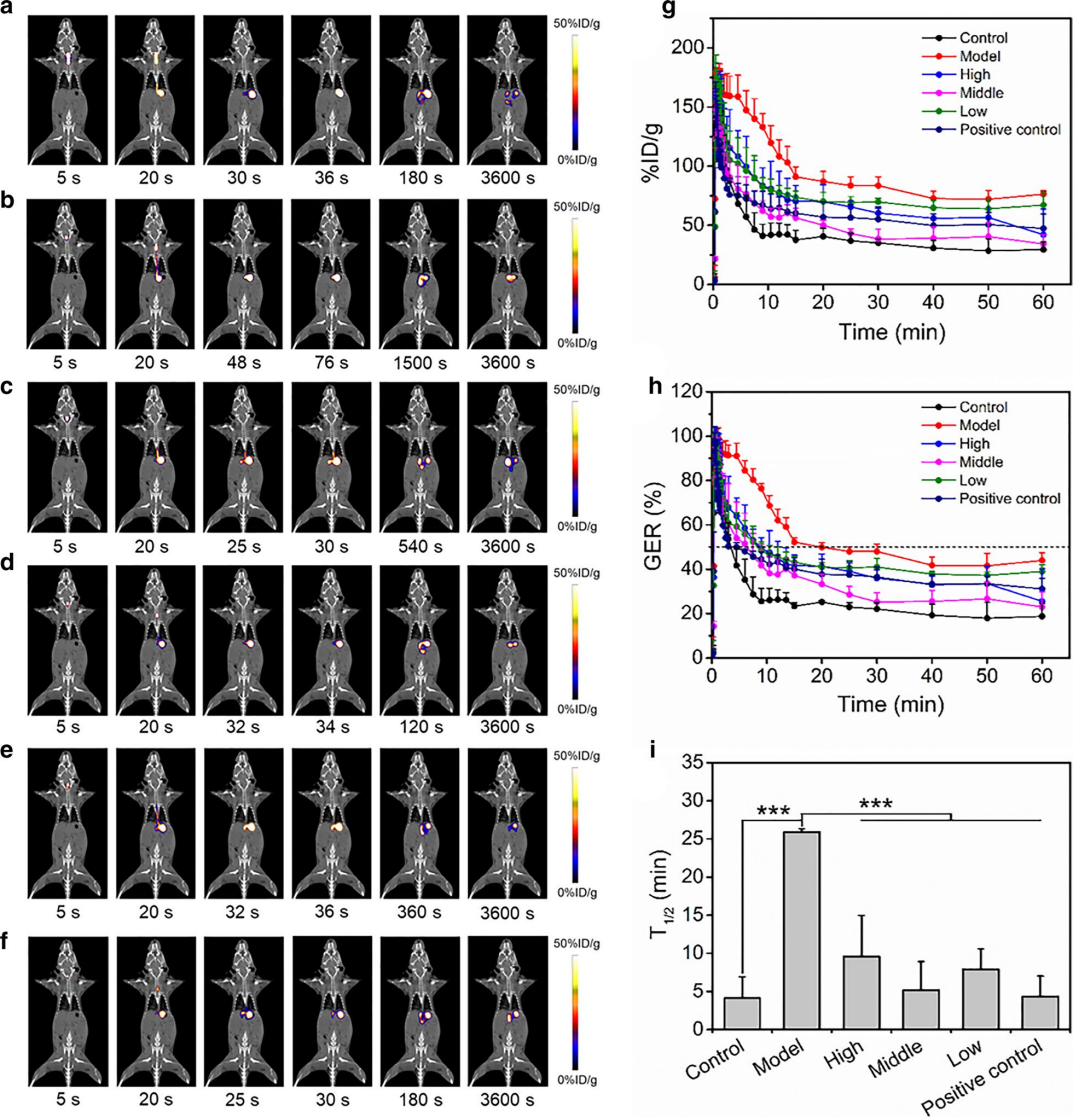
PET evaluation of MCD therapy for constipation. Representative PET images of control group (**a**), model group (**b**), high dose group (**c**), middle dose group (**d**), low dose group (**e**), and positive control group (**f**). The %ID/g (**g**), GER (**h**), and T_1/2_ (**i**) of 6 groups (n = 3/group). ***p < 0.001, and *p* values in **i** were calculated by one-way ANOVA with Bonferroni post-hoc test. *GER* gastric emptying rate

The representative PET images were shown in Fig. 6a–f. The time point that radiotracer began to be discharged from the stomach were earlier in control mice and constipation mice with treatments than untreated constipation mice. Control group had the fastest declines in GER, while untreated constipation group had the slowest reductions. The GER of treatment groups also decreased faster than untreated constipation group (Fig. 6g, h, Additional file 4: Figure S4). As shown in Fig. 6i, the T_1/2_ for control, high, middle, low doses of MCD, and magnesium sulfate group were 4.13 ± 2.78 min, 9.60 ± 5.38 min, 5.20 ± 3.72 min, 7.91 ± 2.67 min, and 4.33 ± 2.70 min, respectively. They were all significantly different from constipation group (25.9 ± 0.41 min). Besides, no significant differences existed among the 3 MCD treatment groups and positive control group, indicating that MCD could ameliorate GE at low doses.

## Discussion

In this study, a noninvasive PET imaging method was established for objective GE visualization and quantification. The radiotracer [^68^Ga]Ga-NOTA had promising purity and stability and was easy to synthesize. The acquired images showed high quality and the quantification method was reliable and easy-to-perform. Since gastrointestinal motility disorders cannot be distinguished from inflammation or malignant diseases based on symptoms and medical history, and abnormal GE can influence and be influenced by many disease processes [42, 43], this established PET method for GE measurement would be promising to satisfy research need.

As a functional imaging modality, PET owns high quantification capability and image quality [19, 20]. PMOD is a widely-used kinetic modeling software for PET and is currently one of the most reliable methods for PET quantification. However, its operating and economic costs are high, especially for research institutions outside of hospitals and which focus on animal experiments. Several studies are devoted to developing quantitative tools that are easier to operate and comparable to PMOD [44–46]. The economic cost of ROI-based analysis is low, and its operation is simple. However, it is questionable whether it can accurately quantify GE because GE is a dynamic process, and the detection of GE can be affected by many factors. Therefore, we compare these two methods. The results showed that the %ID/g calculated by manually delineated ROI was highly correlated with volume and total radioactivity calculated by PMOD, which reflected the GE process well. In other words, the method of manually drawing ROI can substitute the more accurate processing method to reach the purpose, thereby reducing the economic, operation, and time burdens caused by complex quantification.

Although PET has superior sensitivity and quantification capability as well as high image quality and temporal resolution [19], it has not been explored for GE measurement. The primary concerns were its cost-effectiveness and radiation exposure. In this study, we selected ^68^Ga to construct our radiotracer. ^68^Ga is produced by the generator, which can greatly reduce the economic, site, operation, and radiation shielding burdens from the use of the cyclotron [47]. Besides, the tracer [^68^Ga]Ga-NOTA we used in this study can be easily synthesized within 20 min and without further purification. These advantages would improve the cost-effectiveness of PET for GE measurement. In addition, the shorter half-life of ^68^Ga (67.8 min) compared to ^99m^Tc (6.02 h) and ^18^F (110 min) would reduce the exposure duration. Moreover, the high image quality shown in this study makes it possible to flexibly decrease the dosage of radiotracer. These characteristics could reduce the radiation exposure to the subject.

It is generally recognized that PET is a powerful tool for diagnosis and treatment development. Another application of PET is to visualize and quantify markers of pathophysiology [48]. In this study, we used this established PET method to evaluate the efficacy of MCD on delayed GE caused by constipation. The results showed that PET objectively quantified the GE process after MCD treatment and indicated that MCD can improve the GE in constipation at low dosage. In addition, this study also showed that MCD had the ability to ameliorate constipation. Although lots of TCMs showed efficacy for the treatment of gastrointestinal motility disorders, only a few of the underlying mechanisms of these treatments have been explored [49]. Among them, many studies chose to measure the changes in GE during treatment to demonstrate their efficacy [50–52]. The feasibility and availability of PET for GE measurement established in this study would provide a powerful tool for these pharmacological studies.

Our study also has some limitations. Firstly, we did not conduct scintigraphy scans on animal-used equipment. Our data showed the stable visualization and quantification ability and promising potential of PET for the evaluation of GE. Further experiments were needed to directly compare the differences between PET and scintigraphy in GE measurement. Besides, due to the limitation of microPET equipment used in this study, the CT images of mice were manually overlapped on PET images, which might impair some image information. However, we believe that the direct fusion of PET and CT images will further demonstrate the excellent image quality and quantification capability of PET. Finally, the mechanism of MCD in relieving constipation was not clear. Whether the increased rate of GE in MCD was due to the nutritive value of MCD such as its oily components or the medicinal activity of MCD cannot be demonstrated based on existed data. Further pharmacological studies are needed to explore this issue.

## Conclusions

In summary, this study established a noninvasive PET imaging method for GE measurement in mice. The powerful quantification and visualization capabilities of PET made it a unique and important value for evaluating GE. Besides, the more cost-effective radiotracer and quantification method we explored overcame its shortcomings and made it more convenient for applications. The improvement of GE in constipation during MCD treatment was demonstrated by PET. These results demonstrated the feasibility and potential of PET for GE measurement.

## Supplementary Information

**Additional file 1:** Quantification of the first 3 min of dynamic PET images in 4 groups of mice (n = 4/group).

**Additional file 2:** Results of scintigraphy.

**Additional file 3: **Fecal conditions of the mice.

**Additional file 4:** Quantification of the first 1.5 min of dynamic PET images in 6 groups of mice after treatment period (n = 3/group).

## Data Availability

The datasets used and analyzed during the current study are available from the corresponding author on reasonable request.

## Abbreviations

GE: Gastric emptying
MRI: Magnetic resonance imaging
PET: Positron emission tomography
TCM: Traditional Chinese medicine
MCD: Maren chengqi decoction
ROI: Region of interest
GER: Gastric emptying rate
TAC: Time-activity curve
T_1/2_: Half of GE time
CT: Computed tomography
SPECT: Single-photon emission computed tomography
