# Supplementary material for: 68Ga-NOTA PET imaging for gastric emptying assessment in mice
Source: BMC Gastroenterol. 2021 Feb 13;21:69. doi: 10.1186/s12876-021-01642-7 (PMC7881688; doi:10.1186/s12876-021-01642-7)
Supplement: Supplementary file 1 — Additional file 1: Quantification of the first 3 min of dynamic PET images in 4 groups of mice (n = 4/group). [file 12876_2021_1642_MOESM1_ESM.docx]

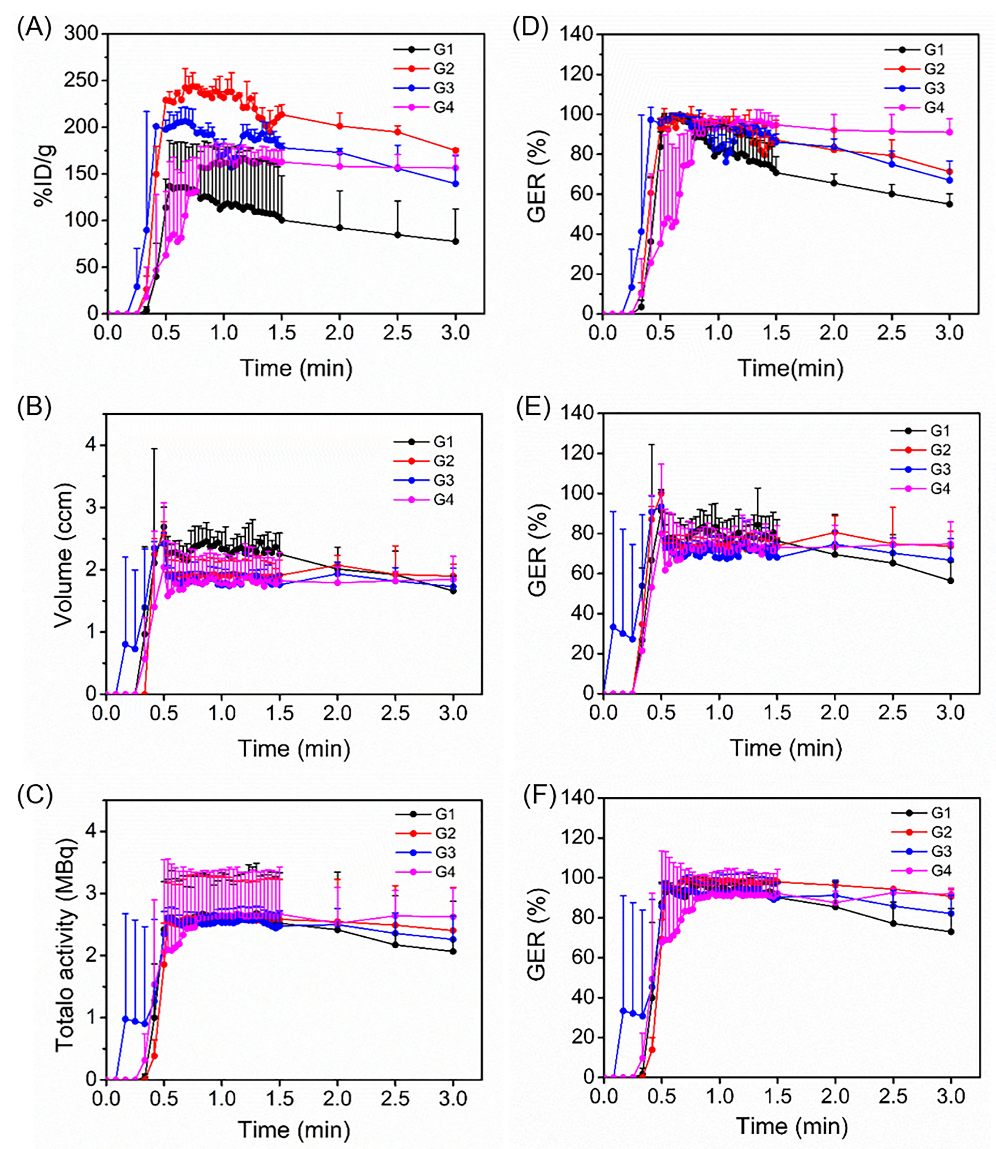


**Additional file - Figure S1** Quantification of the first 3 min of dynamic PET images in 4 groups of mice (n = 4/group). The %ID/g (A) and its derived GER (D). The volume (B) and its derived GER (E). The total radioactivity (C) and its derived GER (F). GER: gastric emptying rate
