# Supplementary material for: 68Ga-NOTA PET imaging for gastric emptying assessment in mice
Source: BMC Gastroenterol. 2021 Feb 13;21:69. doi: 10.1186/s12876-021-01642-7 (PMC7881688; doi:10.1186/s12876-021-01642-7)
Supplement: Supplementary file 2 — Additional file 2: Results of scintigraphy. [file 12876_2021_1642_MOESM2_ESM.docx]

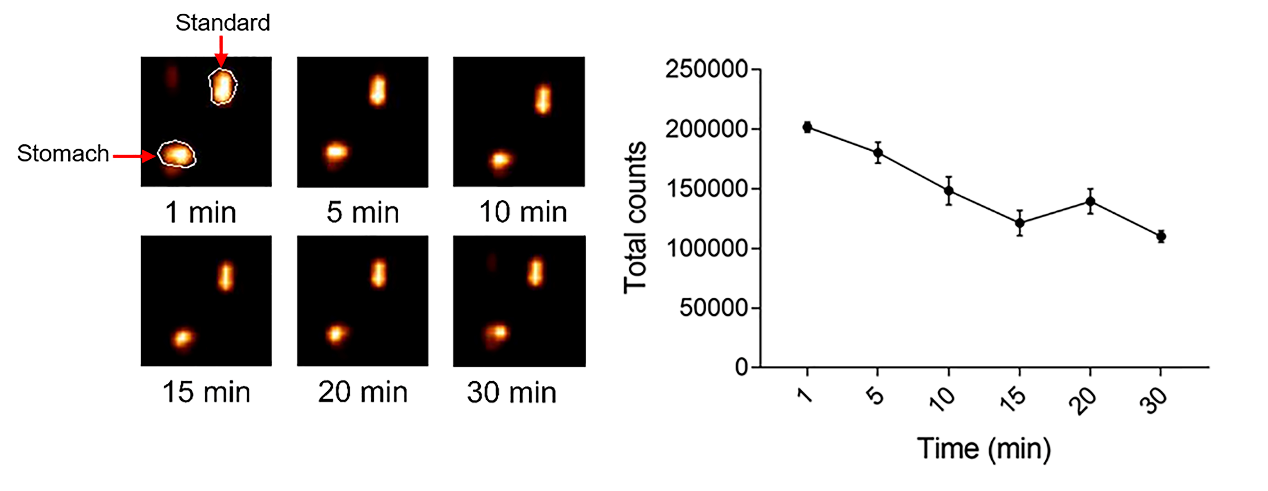


**Additional file - Figure S2** Results of scintigraphy. Representative images (left) and the quantification (right)
