# Supplementary material for: 68Ga-NOTA PET imaging for gastric emptying assessment in mice
Source: BMC Gastroenterol. 2021 Feb 13;21:69. doi: 10.1186/s12876-021-01642-7 (PMC7881688; doi:10.1186/s12876-021-01642-7)
Supplement: Supplementary file 3 — Additional file 3: Fecal conditions of the mice. [file 12876_2021_1642_MOESM3_ESM.docx]

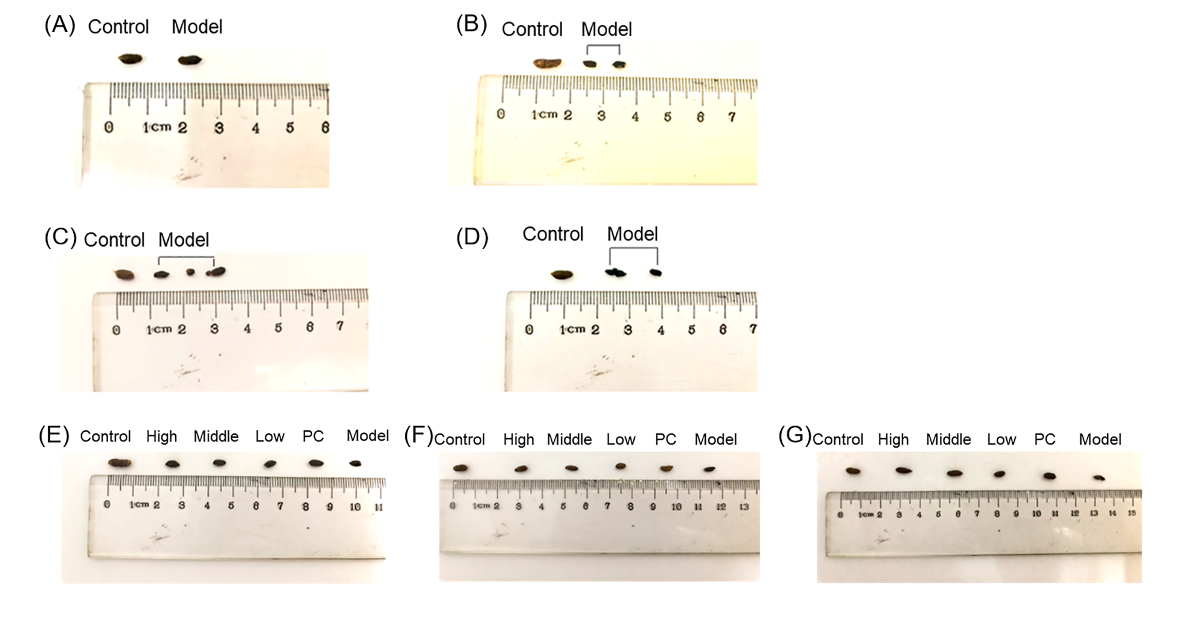


**Additional file - Figure S3** Fecal conditions of the mice. Feces of control and model mice in day 1 (A), day 5 (B), day 7 (C), and day 10 (D) of modeling period. Feces of mice in 6 groups in day 1 (E), day 3 (F), and day 5 (G) of treatment period. PC: positive control
