# Supplementary material for: 68Ga-NOTA PET imaging for gastric emptying assessment in mice
Source: BMC Gastroenterol. 2021 Feb 13;21:69. doi: 10.1186/s12876-021-01642-7 (PMC7881688; doi:10.1186/s12876-021-01642-7)
Supplement: Supplementary file 4 — Additional file 4: Quantification of the first 1.5 min of dynamic PET images in 6 groups of mice after treatment period (n = 3/group). [file 12876_2021_1642_MOESM4_ESM.docx]

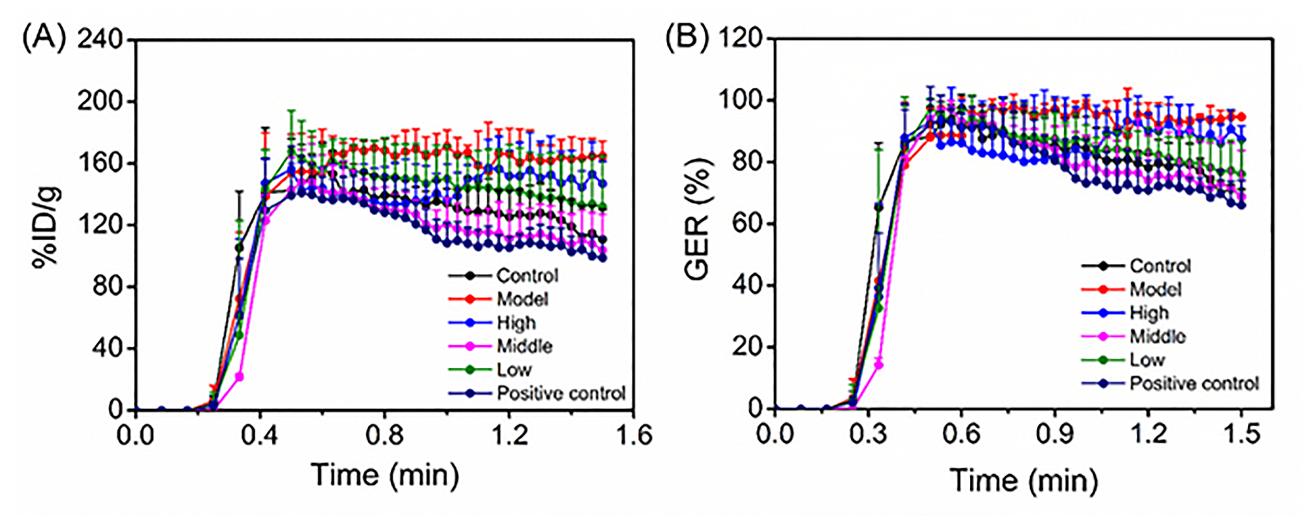


**Additional file - Figure S4** Quantification of the first 1.5 min of dynamic PET images in 6 groups of mice after treatment period (n = 3/group). The %ID/g (A) and its derived GER (B) calculated by manually delineated ROIs. GER: gastric emptying rate
